# Usability of an Educational Intervention to Overcome Therapeutic Inertia in Multiple Sclerosis Care

**DOI:** 10.3389/fneur.2018.00522

**Published:** 2018-07-10

**Authors:** Gustavo Saposnik, Philippe N. Tobler, Fernando Caceres, Maria A. Terzaghi, Christian Ruff, Jorge Maurino, Manuel Fruns Quintana, Jiwon Oh, Xavier Montalban, Muhammad Mamdani

**Affiliations:** ^1^Department of Medicine, Division of Neurology, St. Michael's Hospital, University of Toronto, Toronto, ON, Canada; ^2^Laboratory for Social and Neural Systems Research, Department of Economics, University of Zurich, Zurich, Switzerland; ^3^Decision Neuroscience Unit, Li Ka Shing Knowledge Institute, St. Michael's Hospital, University of Toronto, Toronto, ON, Canada; ^4^Department of Neurology, Instituto de Neurociencias Buenos Aires, Buenos Aires, Argentina; ^5^Neuroscience Area, Medical Department, Roche Farma, Madrid, Spain; ^6^Clinica Las Condes, Santiago, Chile; ^7^Neurology-Neuroimmunology Department and Neurorehabilitation Unit, Multiple Sclerosis Centre of Catalonia (Cemcat), Barcelona, Spain; ^8^Healthcare Analytics Research, Li Ka Shing Knowledge Institute, St. Michael's Hospital, University of Toronto, Toronto, ON, Canada

**Keywords:** multiple sclerosis, educational intervention, usability, decision making, treatment, score

## Abstract

**Background:** Educational interventions are needed to overcome knowledge-to-action gaps in clinical care. We previously tested the feasibility and potential efficacy of an educational intervention that facilitates treatment decisions in multiple sclerosis care. A demonstration of the usability of such an intervention is crucial prior to demonstration of efficacy in a large trial.

**Objectives:** To evaluate the usability of a novel, pilot-tested intervention aimed at neurologists to improve therapeutic decisions in multiple sclerosis (MS) care.

**Methods:** We surveyed 50 neurologists from Chile, Argentina, and Canada randomized to an educational intervention arm of a pilot feasibility study using the System Usability Score (SUS) to assess the usability of a traffic light system (TLS)-based educational intervention. The TLS facilitates therapeutic decisions, allowing participants to easily recognize high-risk scenarios requiring treatment escalation. The SUS is a validated 10-item questionnaire with five response options. The primary outcome was the average and 95% confidence interval (CI) of the SUS score. Values above 68 are considered highly usable.

**Results:** Of 50 neurologists invited to be part of the study, all completed the SUS scale and the study. For the primary outcome, the average usability score was 74.7 (95%CI 70.1–79.2). There was one outlier with a score of 35. The usability score excluding the outlier was 76.8 (95%CI 72.7–80.8). Multivariate analysis revealed no association between participants' characteristics and the SUS score.

**Conclusions:** Our educational intervention has shown high usability among neurologists. The next step is to evaluate the effectiveness of this educational intervention in facilitating treatment decisions for the management of multiple sclerosis in a large trial.

## Background

Recent studies have shown many patients with multiple sclerosis (MS) remain undertreated (1, 2). Therapeutic inertia (TI) is defined as the lack of treatment initiation or escalation when treatment goals are unmet (3, 4). Approximately 70–80% of neurologists caring for patients with MS are estimated to be affected by TI (2). Physician factors are most commonly related to TI (e.g., low tolerance to uncertainty, status quo bias) (5, 6).

Educational strategies are developed in medicine to either fill knowledge gaps (where there is limited background information on the management of a medical condition) or knowledge-to-action gaps (i.e., failure to integrate background knowledge into a diagnostic or therapeutic decision). To the best of our knowledge, there are no such interventions aimed at influencing clinician decisions to ameliorate the consequences of TI (e.g., poor patient outcomes) (2–4). In a previous study, we showed the feasibility and potential efficacy of an educational intervention using a traffic light system (TLS) to overcome TI in the management of MS (7). The TLS emerged as a warning and risk categorization strategy to reduce human errors (8). It facilitates the decision-making process relying on a “hard wired” process that applies the traffic light colors to match three types of situations: red light (“high risk”/“stop and think”), yellow light (“warning”/“reassess in a period of time”) and green light (“stable”/“continue the same strategy”).

The TLS aids the integration of specific situations with an action (9, 10). For example, studies have shown that the TLS can help physicians select the course of action for children presenting with fever or facilitate healthier choices by signaling with a red color food with high content of sugar, cholesterol, and sodium (11). However, many educational interventions or scores are not widely used or implemented in clinical practice. Consequently, it is crucial to determine the potential usability of an educational intervention prior to testing its efficacy in large and expensive randomized clinical trials.

In the present study, we evaluated the usability of our educational intervention among neurologists caring for MS patients. We hypothesized that our educational intervention using the TLS has high usability, as defined by the system usability score (SUS), in this population.

## Methods

**Figure 1:**
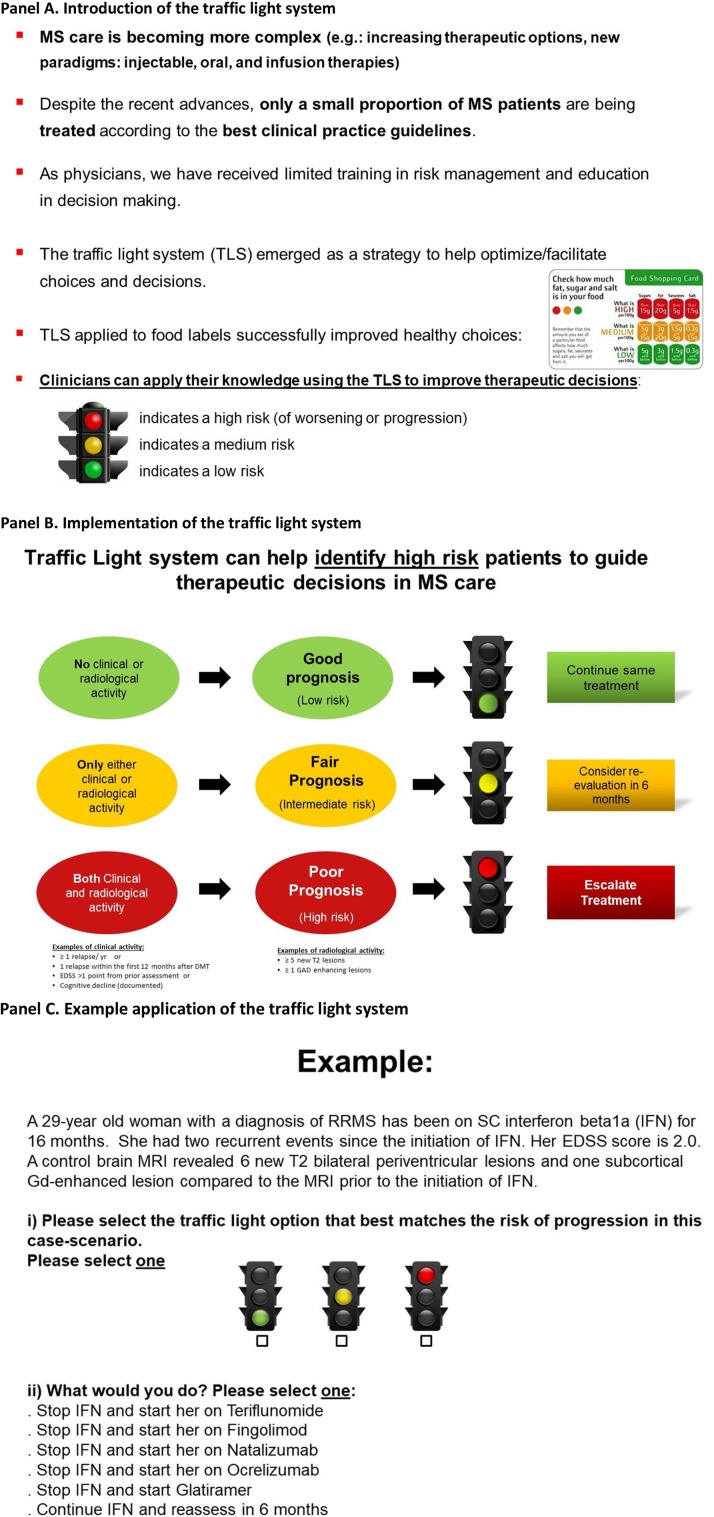
Educational intervention: the traffic light system may facilitate therapeutic decisions in MS care (7). Participants viewed the two informative panels **(A,B)** and a third panel providing an example **(C)**. **(A)** Introduction of the traffic light system. **(B)** Implementation of the traffic light system. **(C)** Example application of the traffic light system.

We included neurologists participating in an international (Chile, Argentina, Canada), parallel-group, randomized pilot study evaluating the feasibility of the educational intervention (TLS: active group) (Figure 1) compared to usual care (control group) in the management of MS (7). The recruitment of participants was facilitated by the National Neurological Societies (MS Society for Canada) of the three participating countries providing mailing lists of non-specialized neurologists and those with MS expertise. Participants randomized to the educational intervention were exposed to 10 case-scenarios and asked to make a therapeutic choice, guided by the TLS-based education intervention. Following these case-scenarios, they were asked to assess the utility of the TLS using the SUS. Given our previous findings (e.g., association between physicians' risk preferences and aversion to ambiguity with TI), we also included previously tested experiments to assess participants' risk preferences and aversion to ambiguity to evaluate their association with the SUS (2). In brief, participants were asked about the minimal certain payoff they would prefer over the equiprobable gamble of winning 400 or 0 US$ (expected value of 200 US$). Ambiguity aversion is defined as dislike for events with unknown probability over events with known probability. Participants had to choose between two options: (a) a 50% chance of winning 400 vs. 0 US$ or (b) an unknown probability of winning 400 US$ (where the unknown probability of winning may be higher or lower than 50%). Further details of the protocol were published in ClinicalTrials.gov # NCT03134794 and elsewhere (2, 7).

### The system usability score (SUS)

The SUS is a validated 10-item questionnaire with a 5-point Likert rating scale that ranges from 1 = strongly disagree to 5 = strongly agree. Questions with positive and negative connotation are alternated to avoid any biases. A score is calculated by subtracting participants' responses from 5 for even questions and by subtracting one from responders' answers for odd questions. This procedure scales all values from 0 to 4 (with four being the most positive response). The final score is obtained by adding the converted responses for each participant and multiplying the total by 2.5. As a result, the possible values range from 0 to 100 (12). A score of 68 or higher is considered “highly acceptable” (13–16). The SUS has been validated in different populations and countries and used in over 500 instruments (13–16).

### Educational intervention (Figure 1): the TLS

Our educational intervention is based on the application of the TLS to medical-decision making (9, 10, 17). The TLS was intended to assist participants in identifying high-risk case-scenarios, where MS patients had both clinical relapses and evidence of radiological activity. Our educational intervention focused on MS because of tiered therapeutic options and consensus about treatment escalation (e.g., presence of clinical relapses and radiological activity) (18, 19). Our educational intervention was delivered in a single presentation. The total duration of the educational intervention was <5 min (Figure 1) (7).

**Figure 2:**
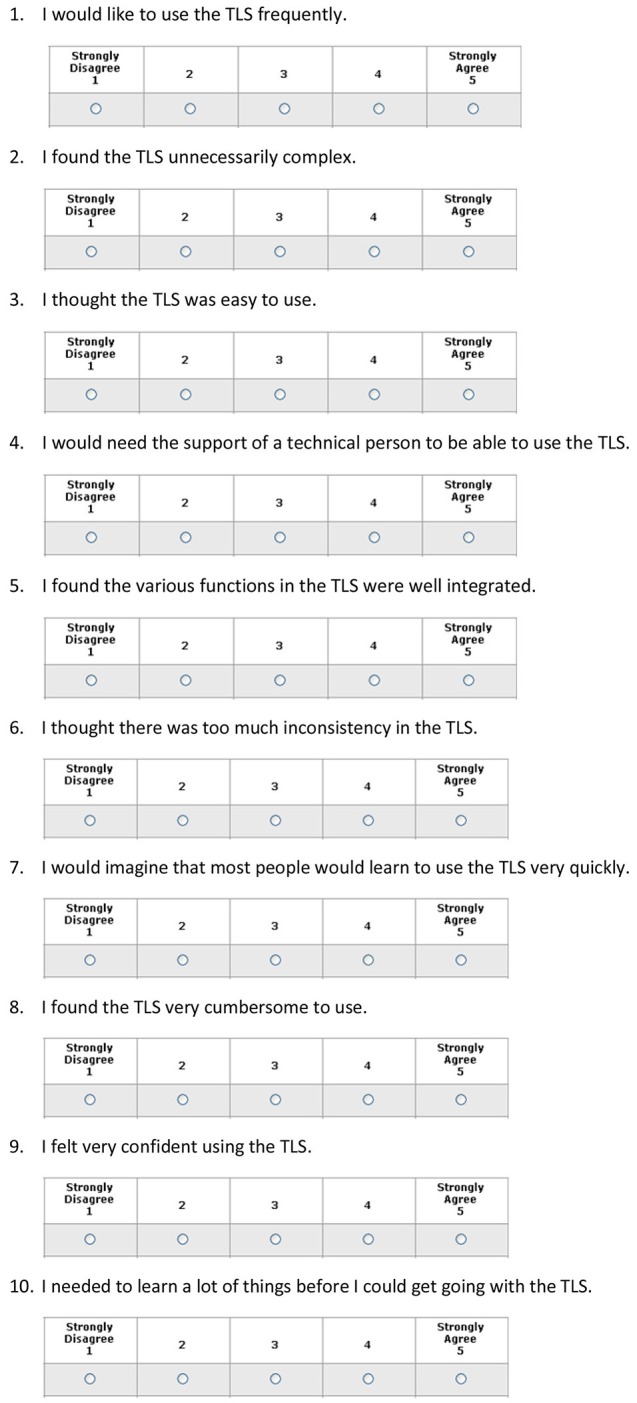
Usability Score System. The SUS is a 10 point-item questionnaire with 5 response options.

Participants were asked to select the traffic light that best matched the case-scenario based on clinical relapse history and results from brain imaging. For example, a “red” traffic light may have been selected for a patient who had both a clinical relapse and some level of activity in brain imaging (Figure 2), where treatment escalation is likely the correct decision. Similarly, participants may have selected the color “yellow” (representing caution requiring a reassessment within 6–12 months) when case-scenarios had either a clinical relapse or some degree of activity in brain imaging (but not both). At the end of the study, participants were asked to evaluate the delivered educational intervention using the validated SUS instrument. Further details were published in the pilot study (7).

### Treatment options

The treatment options for relapsing-remitting MS (RRMS) include first-line (beta interferons, glatiramer acetate, etc.), second-line (fingolimod), and third-line (natalizumab, alemtuzumab) therapies (20). Other recently approved agents were not included if they were not available in the participating countries at the time of the study (e.g., Ocrelizumab, Cladribine, etc.). For the present analysis, we used the aforementioned scheme according to the current clinical practice (2, 21, 22).

### Outcome measures

The primary outcome was the average SUS. Values >68 points are considered to reflect high usability (13, 14). The secondary outcome was the proportion of participants with a usability score >68 points.

### Statistical analysis

Given the pilot nature of this study, we performed primarily descriptive statistics for the primary and secondary outcomes. As a secondary analysis, we developed a linear regression model to explore factors associated with the usability score including age, gender, general neurologist vs. MS specialist status, years of experience, practice setting and volume, physicians' risk preferences and aversion to ambiguity, and authorship in a recent peer-review publication. Variables entered into the models were defined a priori based on the results of our previous studies (e.g., MS specialist vs. general neurologists, physicians' risk preferences and aversion to ambiguity) adjusted for age and sex (7). Specialist status was self-defined. Collinearity among variables was explored by variance inflation factors (VIFs). Values below 10 are considered a good indicator of lack of collinearity. All tests were 2-tailed, and *p* < 0.05 was considered significant. We used STATA 13 (College Station, TX: StataCorp LP) to conduct all analyses.

The study was approved by the Research Ethics Board of St. Michael's Hospital, University of Toronto, Canada.

## Results

Of 50 neurologists who were invited to participate in the study, all (100%) completed the usability scale. Overall, the mean (SD) age was 45.6 (± 11.1) years; 21 (42%) were female neurologists. One third of participants primarily focused their practice on MS care. Table 1 summarizes baseline characteristics of the study population.

**Table 1:** Baseline characteristics of participants.

**Characteristics**	**Total *n* = 50**
**AGE**	
Years (mean ± SD)	45.6 ± 11.1
**SEX**	
Female	21 (42.0)
**SPECIALTY**	
MS specialists	18 (36.0)
General neurologists who care for MS patients	32 (64.0)
**PRACTICE SETTING**	
Academic	21 (42.0)
Community or private institution	29 (58.0)
**% TIME IN CLINICAL PRACTICE**	
50–75%	18 (36.0)
Greater than 75%	25 (50.0)
**YEARS IN PRACTICE**	
mean (±SD)	19.0 ± 11.0
**SEEN MORE THAN 20 MS PATIENTS PER MONTH**	**34 (68.0)**
**AUTHOR OF A PEER-REVIEWED PUBLICATION IN THE**	**23 (46.0)**
**LAST 12 MONTHS**	

*Numbers in brackets indicate percentages*.

For the primary outcome, the average usability score [95% confidence intervals (CI)] was 74.7 (95%CI 70.1–79.2). There was one outlier with a score of 35. The usability score excluding that outlier was 76.8 (72.7–80.8).

**Figure 3:**
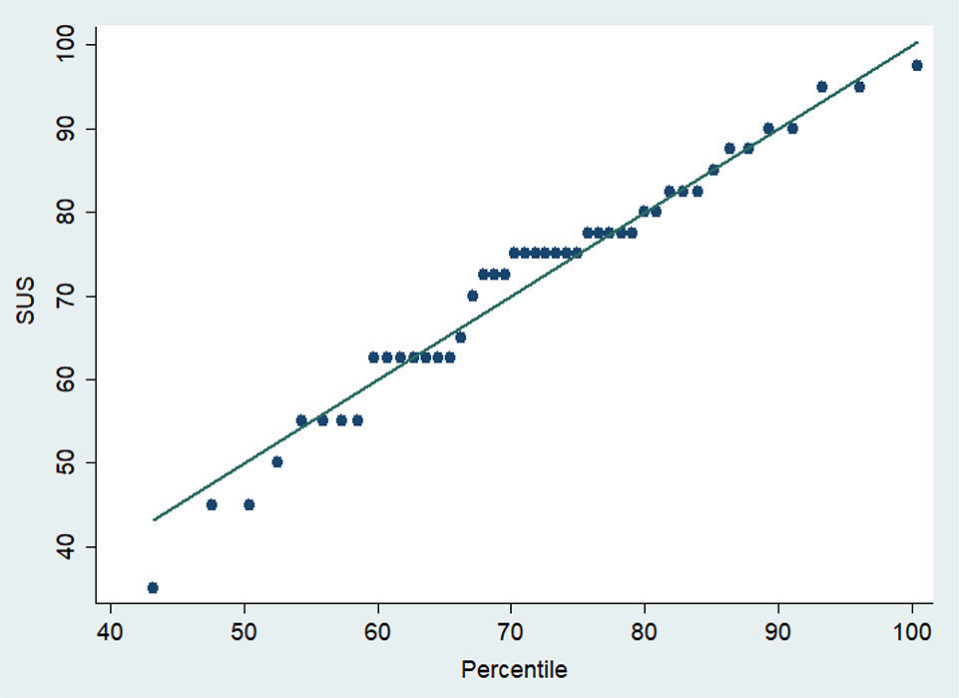
Percentiles of the Usability Score System (SUS).

For the secondary outcome, 70% (*n* = 35) of participants achieved a usability score >68 points. SUS percentiles are shown in Figure 3.

Multivariate regression analysis revealed no statistically significant association between the variables tested and SUS score (Table 2). There was no collinearity among the included variables as reflected by VIF scores < 1.5. Overall, the included variables only explained <9% of the variability of the SUS scores (*r*^2^ = 0.088).

**Table 2:** Multivariate analysis: factors associated with the SUS score.

**Variable**	**Coefficient**	**Standard error**	**95%CI**		**VIF**
Age, in years	0.13	0.22	−0.031	0.58	1.15
Sex	0.50	4.86	−9.30	10.3	1.11
MS specialist (vs. general neurologist)	−6.74	4.84	−16.5	3.02	1.02
Risk preference, in US$	0.014	0.03	−0.04	0.06	1.24
Aversion to ambiguity	−5.44	4.72	−15.0	4.08	1.02
Constant	70.1	10.5	48.8	91.3	NA

*Number of observations: 50; r^2^ = 0.088; NA, not applicable. VIF, Variance inflation factors. Values < 10 are considered as evidence for lack of collinearity*.

## Discussion

In the present study, we found a high level of usability of a TLS-based educational intervention aimed at overcoming TI in the management of MS. Our results suggest that a TLS-based intervention is amenable to being widely used in clinical practice. We identified no specific factors associated with higher usability scores, suggesting that its use may not be affected by age, gender, expertise, years of practice, volume of MS patients seen per week or practice setting. The usability of the TLS was not influenced by individual physicians' risk preferences or aversion to ambiguity.

The use of the TLS is a novel way to optimize treatment decisions. It has been successfully applied to different medical fields, including the selection of healthier food choices leading to weight loss or the detection of children with fever at high risk of developing a serious bacterial infection (9, 10). In a previous randomized trial, we pilot-tested the application of the TLS to determine the feasibility (and preliminary efficacy data for sample size calculation for a larger trial) in treatment decisions in MS care. We showed feasibility and promising results in reducing TI (OR 0.57; 95% CI 0.26–1.22) (7).

Our findings have practical clinical implications. Our results showed excellent usability scores for a simple and brief educational intervention that helps categorize MS patients according to their future risk of disease progression. This educational strategy was designed to ameliorate TI by overcoming knowledge-to-action gaps. Physicians who are resistant to treatment changes (e.g., status quo) when indicated by best practice guidelines may benefit from using the TLS.

Our results nicely integrate with our previous work showing one of the major components influencing TI is aversion to risk and ambiguity (defined as the probability of an event being unknown) (2, 7). To the best of our knowledge, there are no other proven educational interventions to address this phenomenon. Aversion to risk and ambiguity are concepts derived from studies in behavioral economics to characterize individuals and describe their decisions. We previously showed that aversion to ambiguity is an independent predictor of physicians' TI (2). The use of the TLS may help such participants to make prompt decisions and overcome TI.

Our study has some limitations. First, the sample size is small given the pilot design. Second, we used a single measurement of usability. However, the SUS has been validated and extensively applied to over 500 interventions (13–15). Finally, we included participants from three specific countries. It is possible that these findings may not be generalizable to clinicians from other countries.

Despite these limitations, our study suggests that a simple and brief educational intervention applying the TLS may have high potential for use by neurologists when evaluating therapeutic choices for MS patients. Our findings should be put into perspective as the TLS may facilitate early identification of high-risk patients that require a therapeutic change. For example, clinicians may apply the TLS for patients waiting to be seen in the outpatient clinics by adding a color tag to those screened as high-risk according to the medical situation (“red color tag” for MS patients with new symptoms suggestive of a clinical relapse and radiological progression). This approach may help planning resources, facilitate timely discussions and prioritized clinical outcomes when treatment escalation is indicated (23). Although our intervention was designed to facilitate therapeutic decisions in MS care, it can be applied to decisions in other neurological fields (e.g., epilepsy, migraine) and chronic conditions (e.g., hypertension, diabetes). Given that we have tested the feasibility and usability of this promising educational intervention, the next step is the assessment of its efficacy in affecting management decisions and resultant clinical outcomes in a large, properly designed randomized clinical trial.

## Ethics statement

This study was carried out in accordance with the recommendations of the Research Ethics Board of St. Michael's Hospital, University of Toronto, Canada. The protocol was approved by the Research Ethics Board at St Michael's Hospital. All subjects gave informed consent in accordance with the Declaration of Helsinki.

## Author contributions

GS: study concept and design, creation of the educational intervention, acquisition of data, analysis and interpretation of the data, and obtaining funding. PT and CR: study concept and design, interpretation of the data, critical revision of the manuscript for intellectual content, and study supervision. FC: interpretation of the data, and critical revision of the manuscript for intellectual content. MT: study implementation, interpretation of the data, and critical revision of the manuscript for intellectual content. JO and MF: interpretation of the data, critical revision of the manuscript for intellectual content. JM: study concept and design, interpretation of the data, critical revision of the manuscript for intellectual content. XM: supervision of MS case-scenarios, interpretation of the data, critical revision of the manuscript for intellectual content. MM: study concept and design, design of the educational intervention, interpretation of the data, critical revision of the manuscript for intellectual content.

### Conflict of interest statement

PT and CR were funded by the Swiss National Science Foundation (PNT: PP00P1_150739, CRSII3_141965, and 100014_165884, CCR: 105314_152891, CRSII3_141965, and 320030_143443). GS is supported by the Heart and Stroke Foundation Career Award following a peer-reviewed open competition. JM was employed by company Roche Pharma. The remaining authors declare that the research was conducted in the absence of any commercial or financial relationships that could be construed as a potential conflict of interest.
